# Systemic inflammation markers independently associated with increased mortality in individuals with hyperuricemia: Results from the NHANES prospective cohort study

**DOI:** 10.1002/iid3.70032

**Published:** 2024-10-01

**Authors:** Tian Ren, Erye Zhou, Jian Wu, Chao Wang, Yufeng Yin

**Affiliations:** ^1^ Department of Rheumatology and Immunology The First Affiliated Hospital of Soochow University Suzhou Jiangsu China; ^2^ Department of Epidemiology and Biostatistics Beijing Research Institute of Traumatology and Orthopaedics, Beijing Jishuitan Hospital Beijing China

**Keywords:** Inflammation < Processes, Neutrophils < Cells, Innate lymphocytes/Lti < Cells, Monocytes/Macrophages < Cells

## Abstract

**Background:**

Hyperuricemia is associated with increased systemic inflammation. The systemic immune‐inflammation index (SII) and systemic inflammation response index (SIRI) are novel systemic inflammation markers and prognostic markers. However, no studies have evaluated the association between the SII/SIRI and mortality risk in individuals with hyperuricemia. This study aimed to investigate the predictive value of the SII and SIRI for all‐cause and cardiovascular mortality in a large cohort of hyperuricemia patients.

**Methods:**

We conducted a prospective cohort study using data from the National Health and Nutrition Examination Survey (NHANES) 2001‐2020. Hyperuricemia was defined as serum uric acid (SUA) levels of ≥7 mg/dL in men and ≥6 mg/dL in women. The SII and SIRI were calculated based on complete blood count parameters. Associations with all‐cause and cardiovascular mortality were analyzed using Cox proportional hazards models. Nonlinearity and effect modification were assessed using restricted cubic splines (RCS) and interaction analysis.

**Results:**

Among the 6181 participants with hyperuricemia aged 20 years and older, over a total 181 months of follow‐up, there were 936 all‐cause deaths, of which 195 were cardiovascular mortality. In the fully adjusted models, the hazard ratios (HRs) were 1.73 (95% CI 1.42‐2.13) for the SII and 2.18 (95% CI 1.82‐2.62) for the SIRI with all‐cause mortality. The adjusted HRs were 2.08 (95% CI 1.37‐3.14) for the SII and 2.32 (95% CI 1.56‐3.45) for the SIRI with cardiovascular mortality. Spline models identified nonlinear U‐shaped (SII) and J‐shaped (SIRI) relationships of inflammation markers with mortality.

**Conclusions:**

Elevated SII and SIRI are independent predictors of mortality in hyperuricemia patients. These inflammatory biomarkers may improve risk stratification in this high‐risk population. Further research should evaluate utility in guiding preventive interventions.

## Graphical abstract

1. The study aims to investigate the predictive value of SII/SIRI for all‐cause and cardiovascular mortality in patients with hyperuricemia. 2. Cox proportional hazards models indicates significant associations between SII/SIRI and mortality. 3. RCS analysis suggests that both of the SII and SIRI exhibit nonlinear correlations with mortality, characterized by a U‐shaped curve for the SII and a J‐shaped curve for the SIRI in hyperuricemia patients.

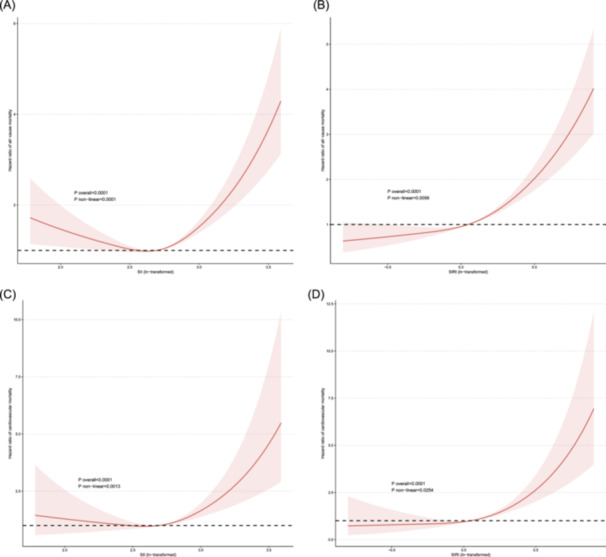

## 1 INTRODUCTION

Hyperuricemia, characterized by elevated serum uric acid (SUA) levels, is a common metabolic disorder affecting hundreds of millions worldwide.^1^, ^2^ In the United States, the occurrence of hyperuricemia has notably risen from 19.1% during the National Health and Nutrition Examination Survey (NHANES) 1988–1994% to 21.5% in NHANES 2007‐2008.^3^ While the prevalence of gout varies significantly, affecting 6% of men and 2% of women, the incidence of hyperuricemia is comparable, at 21.2% for men and 21.6% for women.^3^

Hyperuricemia has been recognized as the major cause of gout, which affects over 8 million adults in America.^4^ In addition, elevated SUA levels have been associated with various metabolic conditions, including hypertension, diabetes, obesity, and metabolic syndrome.^5^ Hyperuricemia has also been linked to increased risks of chronic kidney disease and cardiovascular morbidity and mortality.^6^

Despite the clinical significance, the mechanisms underlying the association between hyperuricemia and adverse health outcomes are not completely understood. Increasing evidence from experimental and clinical studies suggests that chronic low‐grade inflammation may be an important mediator.^7^ SUA has been shown to induce oxidative stress and inflammatory responses in vascular endothelial and smooth muscle cells.^8^ Population studies have found positive correlations of SUA levels with circulating inflammatory cytokines and acute phase proteins such as C‐reactive protein (CRP) and pro‐inflammatory cytokines such as interleukin‐6 (IL‐6) and IL‐1β.^9^ Suppression of inflammation has been proposed as a potential mechanism for the cardioprotective effects of urate‐lowering therapies.^10^

The systemic immune‐inflammation index (SII), based on peripheral lymphocyte, neutrophil, and platelet counts, was originally developed as a prognostic indicator for patients after curative resection for hepatocellular carcinoma.^11^ Recent studies have reported SII as a novel biomarker associated with various metabolic disorders and predictive of mortality in the general population.^12^ The systemic inflammation response index (SIRI), calculated using neutrophil, lymphocyte, and monocyte counts, has also been investigated as a prognostic biomarker for sepsis, cancers, and cardiovascular disease.^13^

However, no studies to date have evaluated the prognostic significance of the SII and SIRI specifically in patients with hyperuricemia, who are at high risk for mortality. Given the potential role of inflammation in hyperuricemia, the SII and SIRI may capture additional predictive information on mortality beyond traditional risk factors in this population.

Therefore, in this large prospective cohort study, we aimed to examine the associations of the SII and SIRI with all‐cause and cardiovascular mortality among US adults with hyperuricemia. We hypothesized that elevated levels of SII and SIRI would be independent predictors of mortality even after adjusting for demographic and socioeconomic confounders. Findings from this study could provide novel evidence to support incorporating the SII and SIRI in risk stratification models among hyperuricemia patients.

## 2 METHODS

### 2.1 Study population

The data source was the NHANES, an ongoing program of studies assessing the health and nutritional status of adults and children in the United States.^14^ The NHANES combines interviews, physical examinations, and laboratory tests using a complex, multistage probability sampling design to provide representative data on the U.S. civilian noninstitutionalized population. All participants provided written informed consent, and the institutional review board of the National Center for Health Statistics (NCHS) approved all procedures.

**Figure 1:**
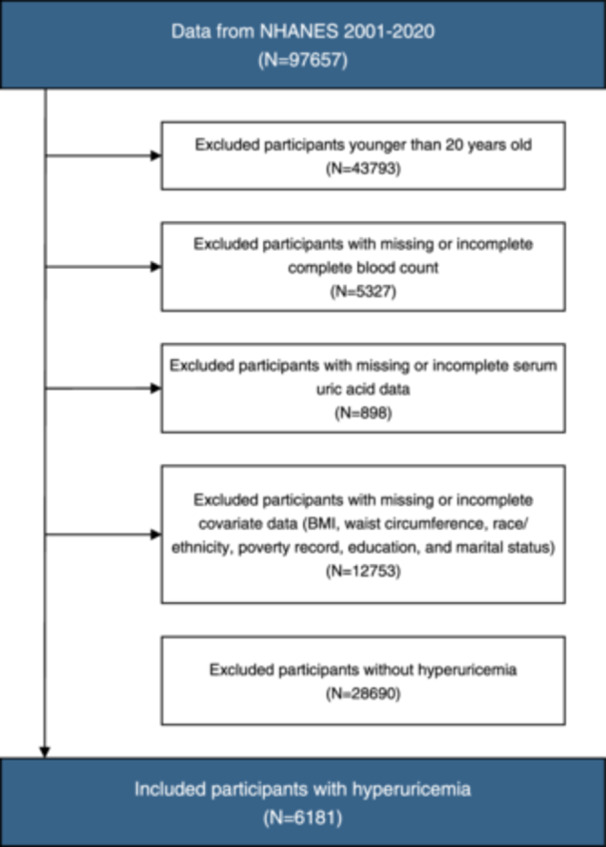
Flow diagram of subjects included in the cohort study.

This analysis included adult participants aged ≥20 years with hyperuricemia between 2001and March 2020 (Figure S1). Hyperuricemia was defined as SUA levels ≥7 mg/dL in men and ≥6 mg/dL in women.^15^ Participants were excluded if they had missing data on uric acid levels, white blood cell differential count, mortality or other covariates included in regression models. The flowchart of inclusion and exclusion is presented in Figure 1.

### 2.2 Mortality outcomes

Participants were followed for mortality status from the date of survey participation through December 31, 2019. Mortality data were obtained through probabilistic linkage between NHANES 2001–2020 participants and National Death Index (NDI) death certificate records. The NDI assigns a probability‐based score to each record matched using social security number, name, sex, race/nationality, date/state of birth, state of death, death certificate number, and date of death. Total mortality included deaths from all causes. Cardiovascular mortality was defined as deaths due to diseases of the circulatory system based on the International Classification of Diseases, 10th Revision codes (I00‐I09, I11, I13, I20‐I51).^16^

### 2.3 Measurement of the SII and SIRI

**Figure 2:**
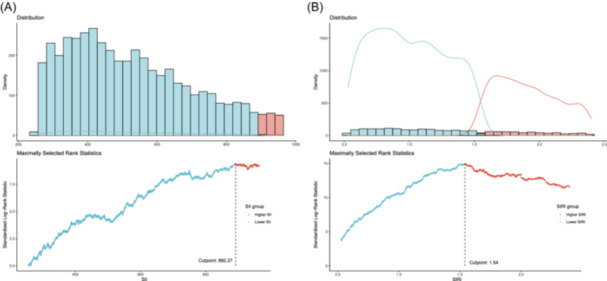
Cutoff values of inflammation markers determined using maximally selected rank statistics method (MSRSM). (A) MSRSM plot identifying the optimal cutoff point for SII in relation to all‐cause mortality in individuals with hyperuricemia. The cutoff value was determined to be 892.27. (B) MSRSM plot identifying the optimal cutoff point for SIRI in relation to all‐cause mortality. The cutoff value was determined to be 1.54. Participants with SII and SIRI levels above these cutoffs were classified as having higher values in subsequent analyses.

The SII was calculated as platelet count × neutrophil count/lymphocyte count, while the SIRI was defined as neutrophil count × monocyte count/lymphocyte count.^11^, ^12^ These were calculated based on complete blood count parameters measured at the Mobile Examination Center (MEC) visit. The maximally selected rank statistics method (MSRSM) was used to determine optimal cutoff points for the SII and SIRI related to all‐cause mortality.^17^ This data‐driven approach identified cutoffs of 892.27 for the SII and 1.54 for the SIRI in participants with hyperuricemia (Figure 2). Participants with values above the cutoffs were classified as having higher SII and SIRI, while those below were classified as lower.

### 2.4 Covariates

Demographic covariates included continuous age, sex (male/female), and race/ethnicity (Non‐Hispanic white, Non‐Hispanic black, Mexican American, other Hispanic, and other/mixed race). Socioeconomic status was evaluated using educational attainment (less than 9th grade, 9–11th grade, high school graduate, some college, college graduate and above) and poverty‐income ratio (<1.29, 1.30‐3.49, ≥3.50). Lifestyle and health factors comprised body mass index (BMI) categories of underweight (<18.5 kg/m^2^), normal (18.5‐24.9 kg/m^2^), overweight (25.0‐29.9 kg/m^2^), or obese (≥30.0 kg/m^2^); smoking status (current smoker vs. nonsmoker); and health insurance status (insured vs. uninsured). Sequential adjustment for potential confounding variables was conducted by first including demographic factors, which was followed by the addition of socioeconomic factors and then lifestyle factors in multivariable Cox regression models.

### 2.5 Statistical analysis

Our analysis rigorously incorporated the complex NHANES survey design, integrating sampling weights (WTMEC4YR, WTMEC2YR, and WTMEPRP) to ensure nationally representative estimates. We utilized the maximally selected rank statistics method (MSRSM) from the ‘maxstat’ package (https://CRAN.R-project.org/package=maxstat)^18^ to determine optimal cutoff points for SII and SIRI related to survival outcomes. Participants were divided into higher and lower SII and SIRI groups based on these cutoffs.

We summarized the baseline characteristics of the study population using means and standard deviations (SDs) for continuous variables and unweighted percentages for categorical variables. Various statistical methods were applied depending on the variable type: analysis of variance (ANOVA) assessed differences in continuous variables among multiple groups, the Wilcoxon rank‐sum test evaluated differences in ordered variables between two groups, and the chi‐square test with Rao & Scott's second‐order correction was used for categorical variables, ensuring accurate analysis in complex survey or clustered data.

Survival probabilities between higher and lower SII/SIRI groups were analyzed using the Kaplan‐Meier method and the log‐rank test. Cox proportional hazards regression estimated hazard ratios (HRs) and 95% confidence intervals (CIs) for associations with all‐cause and cardiovascular mortality. Models were adjusted for potential confounders (age, sex, race/ethnicity, educational attainment, smoking status, BMI, health insurance, and poverty‐income ratio) and explored interactions with prespecified effect modifiers (sex, age group, race/ethnicity, education, and other factors) in subgroup analyses.

**Figure 3:**
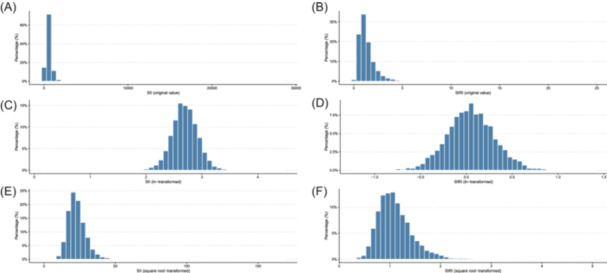
Distribution of inflammation markers with different transformations. (A–C) Histograms showing the distribution of SII in its original, ln‐transformed, and square root‐transformed scales. (D–F) Histograms showing the distribution of SIRI in its original, ln‐transformed, and square root‐transformed scales. The x‐axis displays the SII/SIRI values and y‐axis shows the percentage of participants. The original SII and SIRI both exhibited right‐skewed distributions. After ln‐transformation, SII and SIRI showed adequate normality. Therefore, ln‐transformed values were used for RCS models to assess nonlinearity.

To assess nonlinear dose–response relationships, restricted cubic splines (RCS) with three knots were used. Natural logarithmic and square root transformations were applied to the right‐skewed SII and SIRI values for normalization. The ln‐transformed SII and SIRI values, exhibiting adequate normality, were employed in all RCS models (Figure 3).

## 3 RESULTS

### 3.1 Study population

The final analytical sample consisted of 6181 individuals diagnosed with hyperuricemia. Figure S2 display the distribution of patients with hyperuricemia across different NHANES cycles based on sex and age groups. Table 1 presents the weighted baseline characteristics of the participants with hyperuricemia. The mean age was 50.46 years (SD 17.43), and 57.46% were male. The study population comprised predominantly Non‐Hispanic whites (72.76%), followed by Non‐Hispanic blacks (11.71%), Mexican Americans (5.60%), other Hispanics (3.77%) and other/mixed races (6.16%), and 23.77% were college graduates or above. The mean poverty income ratio was 2.96, and 41.01% had income levels ≥3.5 times the poverty threshold. Regarding lifestyle factors, 49.62% were current smokers, and 55.38% were obese. The mean BMI was 32.06 kg/m2, and the mean waist circumference was 107.55 cm. The majority (84.24%) had health insurance coverage. Comparing participants with higher vs lower SII, those with elevated SII were older (mean age 52.27 vs 50.20 years) and had a higher prevalence of smoking and obesity (all *p* < .001). Similar trends were observed for participants with higher SIRI levels compared to those with lower SIRI levels.

**Table 1:** Characteristics of the patients with hyperuricemia according to systemic inflammation indices.

Characteristic	Overall (*n* = 6181)	SII			SIRI		
		Higher SII (*n* = 786)	Lower SII (*n* = 5395)	P value	Higher SIRI (*n* = 1769)	Lower SIRI (*n* = 4412)	*P* value
Weighted No.	56,290,207	7,041,290	49,248,917		16,700,062	39,590,146	
Age (year)	50.46 (17.43)	52.27 (18.68)	50.20 (17.23)	0.029	53.35 (18.77)	49.24 (16.68)	<0.001
Age group				0.005			<0.001
20–39 years	1498 (28.69%)	176 (27.98%)	1322 (28.80%)		365 (26.08%)	1133 (29.80%)	
40–59 years	1765 (34.72%)	185 (29.23%)	1580 (35.50%)		405 (28.72%)	1360 (37.25%)	
60–79 years	2105 (26.33%)	277 (28.82%)	1828 (25.97%)		640 (30.65%)	1465 (24.51%)	
≥80 years	813 (10.26%)	148 (13.96%)	665 (9.73%)		359 (14.56%)	454 (8.44%)	
Sex				<0.001			0.018
Female	2715 (42.54%)	393 (51.35%)	2322 (41.28%)		691 (39.27%)	2024 (43.92%)	
Male	3466 (57.46%)	393 (48.65%)	3073 (58.72%)		1078 (60.73%)	2388 (56.08%)	
Race/ethnicity				0.001			<0.001
Mexican American	749 (5.60%)	94 (5.50%)	655 (5.62%)		174 (4.35%)	575 (6.13%)	
Other Hispanic	376 (3.77%)	57 (4.34%)	319 (3.68%)		104 (3.58%)	272 (3.85%)	
Non‐Hispanic White	3077 (72.76%)	466 (77.81%)	2611 (72.04%)		1134 (80.83%)	1943 (69.36%)	
Non‐Hispanic Black	1486 (11.71%)	124 (7.69%)	1362 (12.28%)		257 (6.50%)	1229 (13.90%)	
Other/multiracial	493 (6.16%)	45 (4.65%)	448 (6.37%)		100 (4.74%)	393 (6.75%)	
Educational attainment				0.706			0.320
Less than 9th grade	657 (5.61%)	73 (5.11%)	584 (5.68%)		186 (5.77%)	471 (5.54%)	
9–11th grade	891 (10.92%)	120 (10.73%)	771 (10.94%)		266 (11.55%)	625 (10.65%)	
High school graduate	1590 (26.62%)	215 (29.13%)	1375 (26.26%)		477 (28.12%)	1113 (25.99%)	
Some college	1850 (33.08%)	239 (31.67%)	1611 (33.29%)		518 (32.58%)	1332 (33.30%)	
College graduate or above	1193 (23.77%)	139 (23.35%)	1054 (23.83%)		322 (21.98%)	871 (24.52%)	
Poverty income ratio	2.96 (1.60)	2.77 (1.59)	2.99 (1.60)	0.009	2.86 (1.58)	3.01 (1.61)	0.020
Poverty income ratio group				0.022			0.072
0–1.29	1859 (20.54%)	251 (24.09%)	1608 (20.03%)		555 (22.31%)	1304 (19.79%)	
1.3–3.49	2447 (38.45%)	327 (39.99%)	2120 (38.22%)		714 (39.57%)	1733 (37.97%)	
≥3.50	1875 (41.01%)	208 (35.92%)	1667 (41.74%)		500 (38.11%)	1375 (42.24%)	
BMI (kg/m^2^)	32.06 (7.19)	32.57 (8.56)	31.98 (6.97)	0.644	32.33 (7.81)	31.94 (6.91)	0.645
BMI group				0.145			0.486
Underweight	27 (0.33%)	8 ( .85%)	19 (0.26%)		10 ( .31%)	17 (0.34%)	
Normal	823 (12.72%)	115 (13.60%)	708 (12.60%)		259 (13.54%)	564 (12.38%)	
Overweight	1928 (31.57%)	234 (29.73%)	1694 (31.83%)		549 (30.09%)	1379 (32.19%)	
Obese	3403 (55.38%)	429 (55.82%)	2974 (55.32%)		951 (56.06%)	2452 (55.09%)	
Waist circumference (cm)	107.55 (15.92)	108.65 (18.02)	107.39 (15.59)	0.338	109.28 (17.07)	106.82 (15.35)	<0.001
Smoking status				0.007			<0.001
Nonsmoker	3090 (50.38%)	355 (43.91%)	2735 (51.30%)		775 (44.07%)	2315 (53.04%)	
Smoker	3091 (49.62%)	431 (56.09%)	2660 (48.70%)		994 (55.93%)	2097 (46.96%)	
Health insurance				0.986			0.099
Insured	5107 (84.24%)	669 (84.26%)	4438 (84.23%)		1517 (85.66%)	3590 (83.63%)	
Uninsured	1074 (15.76%)	117 (15.74%)	957 (15.77%)		252 (14.34%)	822 (16.37%)	

Abbreviations: BMI, body mass index; SII, systemic immune‐inflammation index; SIRI, systemic inflammation response index.

### 3.2 Systemic inflammation markers and mortality

Among all included participants with hyperuricemia, the median follow‐up time was 74 months (IQR 45–124 months). Over a total 181 months of follow‐up, there were 936 all‐cause deaths, of which 195 were cardiovascular mortality. In the group with a higher SII (*n* = 786), 212 all‐cause deaths and 51 cardiovascular deaths occurred over the follow‐up. For SIRI, the higher SIRI group (n = 1,769) had 453 all‐cause and 108 cardiovascular deaths, while the lower SIRI group (n = 4,412) had 483 and 87 events, respectively.

**Figure 4:**
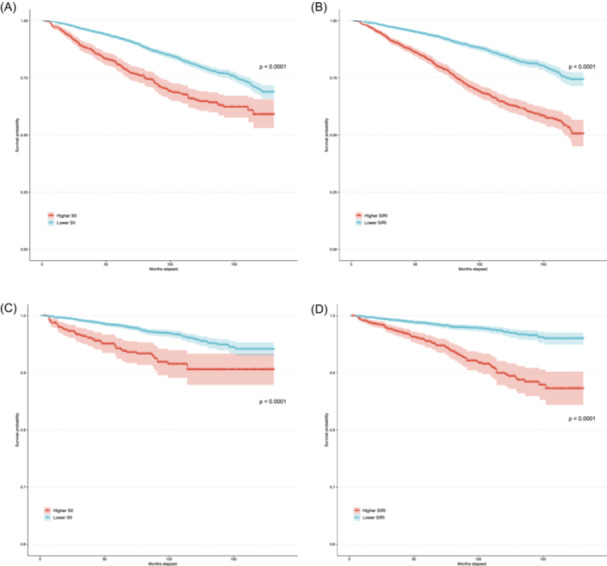
Kaplan‐Meier survival curves for different outcomes and inflammation markers in participants with hyperuricemia. (A, B) Kaplan‐Meier curves for all‐cause mortality stratified by SII and SIRI respectively. (C, D) Kaplan‐Meier curves for cardiovascular mortality based on SII and SIRI, respectively. The x‐axis represents the follow‐up time in months, and the y‐axis represents the cumulative survival probability.

Kaplan‐Meier curves displayed significantly higher all‐cause and cardiovascular mortality among participants with elevated SII and SIRI levels (both log‐rank *p* < .001) (Figure 4). In multivariable Cox regression, after adjusting for demographics, socioeconomic factors and comorbidities, the hazard ratio for each 1‐SD increase in the SII was 1.73 (95% CI 1.42–2.13) for all‐cause mortality and 2.08 (95% CI 1.37–3.14) for cardiovascular mortality. The adjusted hazard ratios for SIRI were 2.18 (95% CI 1.82–2.62) and 2.32 (95% CI 1.56–3.45) for all‐cause and cardiovascular mortality, respectively (Table 2).

**Table 2:** Weighted multivariate logistic analysis of systemic inflammatory indices and mortality in patients with hyperuricemia.

Characteristic	Model 1			__	Model 2			Model 3		
	HR	95% CI	*P* value		HR	95% CI	*P* value	HR	95% CI	*P* value
All‐cause mortality										
SII	1.98	1.62, 2.42	<0.001		1.84	1.49, 2.26	<0.001	1.73	1.42, 2.13	<0.001
SIRI	2.71	2.27, 3.24	<0.001		2.29	1.90, 2.77	<0.001	2.18	1.82, 2.62	<0.001
Cardiovascular mortality										
SII	2.32	1.62, 3.34	<0.001		2.18	1.46, 3.25	<0.001	2.08	1.37, 3.14	<0.001
SIRI	3.15	2.23, 4.44	<0.001		2.46	1.69, 3.60	<0.001	2.32	1.56, 3.45	<0.001

*Note*: Model 1 was unadjusted. Model 2 was adjusted for age, sex, race/ethnicity, and educational attainment. Model 3 was adjusted for age, sex, race/ethnicity, educational attainment, smoking status, BMI, health insurance, and the poverty income ratio.

Abbreviations: BMI, body mass index; HR, hazard ratios; SII, systemic immune‐inflammation index; SIRI, systemic inflammation response index.

### 3.3 Nonlinear regression analysis

**Figure 5:**
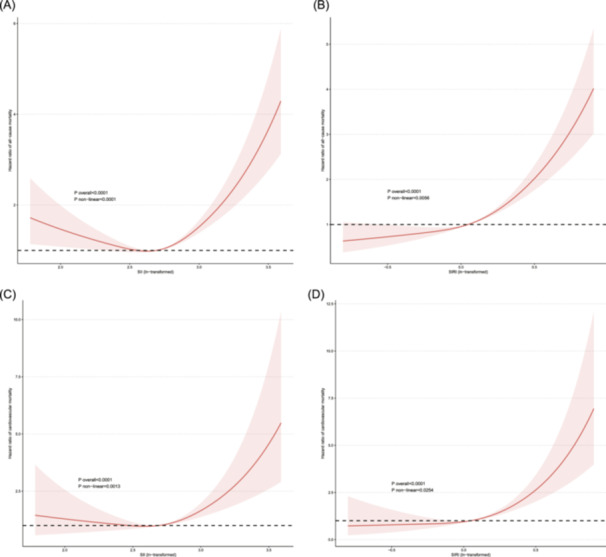
Restricted cubic spline (RCS) analysis of the association between inflammation markers and mortality outcomes. (A, B) The relationship of SII, SIRI and all‐cause mortality. (C, D) The relationship of SII, SIRI and cardiovascular mortality. The x‐axis shows the ln‐transformed values of SII and SIRI. Hazard ratios are plotted on the y‐axis with 95% confidence intervals shown as shaded areas. Nonlinear U‐shaped associations were observed for SII with both all‐cause and cardiovascular mortality. SIRI demonstrated nonlinear J‐shaped relationships with the mortality outcomes.

Nonlinear regression analysis revealed a U‐shaped association between SII and all‐cause mortality (p‐overall <0.0001; p‐nonlinear <.0001), with the lowest risk observed at SII levels of approximately 2.54 on the ln‐transformed scale. For cardiovascular mortality, the SII demonstrated a U‐shaped relationship (P overall <0.0001; P nonlinear=.0013), with the nadir of the curve at an SII of approximately 2.46 (Figure 5A and C).

The association between SIRI and all‐cause mortality was J‐shaped (P overall <0.0001; P nonlinear=.0056), with the risk decreasing at SIRI levels up to 0.054 on the ln‐transformed scale and then increasing steadily afterwards. For cardiovascular mortality, a similar J‐shaped association was observed for SIRI (P overall <0.0001; P nonlinear=.0254), with the lowest risk occurring at SIRI values of approximately 0.054 (Figure 5B and D).

### 3.4 Subgroup analyses and interaction test

Stratified analyses identified that the relationships of elevated SII and SIRI with increased all‐cause and cardiovascular mortality persisted consistently across demographic and clinical characteristics in adults with hyperuricemia (Tables 3 and 4). In most subgroups, higher SII and SIRI were significantly associated with increased risks of all‐cause and cardiovascular mortality, with HRs > 1 and P values < 0.05, aligning with the overall results mentioned above.

**Table 3:** Subgroup analysis of all‐cause mortality of patients with hyperuricemia.

Characteristic	SII			SIRI		
	HR (95% CI)	*P* value	P for interaction	HR (95% CI)	*P* value	P for interaction
Overall	2.07 (1.77, 2.41)	<0.001		2.63 (2.32, 2.99)	<0.001	
Sex			0.336			0.136
Female	1.91 (1.53, 2.39)	<0.001		2.98 (2.45, 3.61)	<0.001	
Male	2.21 (1.79, 2.73)	<0.001		2.43 (2.05, 2.89)	<0.001	
Age group			0.153			0.374
20–59 years	1.47 ( .96, 2.23)	0.074		1.97 (1.43, 2.71)	<0.001	
≥60 years	2.03 (1.72, 2.39)	<0.001		2.32 (2.01, 2.67)	<0.001	
Race/ethnicity			0.753			0.405
White	2.01 (1.68, 2.41)	<0.001		2.6 (2.22, 3.04)	<0.001	
Other	1.91 (1.42, 2.57)	<0.001		2.29 (1.8, 2.9)	<0.001	
Educational attainment			0.656			0.092
Less than 9th grade	1.73 (1.15, 2.61)	0.009		2.08 (1.53, 2.82)	<0.001	
9–11th grade	1.96 (1.37, 2.81)	<0.001		2.34 (1.74, 3.16)	<0.001	
High school graduate	2.18 (1.66, 2.88)	<0.001		2.97 (2.34, 3.78)	<0.001	
Some college	2.28 (1.67, 3.1)	<0.001		2.56 (1.97, 3.34)	<0.001	
College graduate or above	2.52 (1.61, 3.94)	<0.001		3.93 (2.61, 5.9)	<0.001	
Obesity			0.544			0.176
Non‐obesity	1.97 (1.61, 2.41)	<0.001		2.41 (2.04, 2.86)	<0.001	
Obesity	2.18 (1.72, 2.76)	<0.001		2.87 (2.35, 3.5)	<0.001	
Smoking status			0.18			0.47
Nonsmoker	1.78 (1.37, 2.31)	<0.001		2.72 (2.21, 3.34)	<0.001	
Smoker	2.21 (1.83, 2.67)	<0.001		2.46 (2.09, 2.9)	<0.001	
Health insurance			0.088			0.004
Insured	2.12 (1.81, 2.48)	<0.001		2.73 (2.39, 3.12)	<0.001	
Uninsured	1.13 ( .56, 2.28)	0.727		1.25 ( .75, 2.09)	0.396	
Poverty income ratio			0.077			0.011
0–1.29	1.58 (1.19, 2.09)	0.001		1.97 (1.58, 2.46)	<0.001	
1.30–3.49	2.21 (1.78, 2.75)	<0.001		2.85 (2.36, 3.45)	<0.001	
≥3.50	2.43 (1.74, 3.39)	<0.001		3.25 (2.45, 4.3)	<0.001	

Abbreviations: BMI, body mass index; HR, hazard ratio; SII, systemic immune‐inflammation index; SIRI, systemic inflammation response index.

**Table 4:** Subgroup analysis of cardiovascular mortality of patients with hyperuricemia.

Characteristic	SII			SIRI		
	HR (95% CI)	*P* value	P for interaction	HR (95% CI)	*P* value	P for interaction
Overall	2.49 (1.81, 3.43)	<0.001		3.44 (2.59, 4.56)	<0.001	
Sex			0.961			0.991
Female	2.57 (1.54, 4.29)	<0.001		3.34 (2.09, 5.34)	<0.001	
Male	2.54 (1.68, 3.83)	<0.001		3.39 (2.37, 4.84)	<0.001	
Age group			0.152			0.408
20–59 years	3.85 (1.8, 8.23)	0.001		3.8 (1.86, 7.79)	<0.001	
≥60 years	2.09 (1.46, 2.97)	<0.001		2.76 (2.03, 3.75)	<0.001	
Race/ethnicity			0.168			0.603
White	2.63 (1.84, 3.75)	<0.001		3.3 (2.35, 4.63)	<0.001	
Other	1.42 ( .64, 3.15)	0.385		2.76 (1.58, 4.81)	<0.001	
Educational attainment			0.169			0.022
Less than 9th grade	.91 ( .28, 2.97)	0.870		1.46 ( .71, 3)	0.306	
9–11th grade	1.92 ( .78, 4.75)	0.156		2.31 (1.1, 4.85)	0.027	
High school graduate	3.37 (1.91, 5.95)	<0.001		5.05 (2.86, 8.92)	<0.001	
Some college	3.36 (1.85, 6.09)	<0.001		3.61 (2.07, 6.32)	<0.001	
College graduate or above	2.27 (1.01, 5.12)	0.047		6.26 (2.87, 13.67)	<0.001	
Obesity			0.847			0.051
Non‐obesity	2.53 (1.66, 3.84)	<0.001		2.68 (1.85, 3.89)	<0.001	
Obesity	2.39 (1.46, 3.92)	0.001		4.61 (2.97, 7.17)	<0.001	
Smoking status			0.322			0.316
Nonsmoker	1.93 (1.07, 3.46)	0.029		3.98 (2.48, 6.4)	<0.001	
Smoker	2.74 (1.87, 4.02)	<0.001		2.97 (2.09, 4.23)	<0.001	
Health insurance			0.522			0.741
Insured	2.39 (1.71, 3.33)	<0.001		3.41 (2.54, 4.57)	<0.001	
Uninsured	3.51 (1.08, 11.41)	0.037		2.87 ( .97, 8.55)	0.058	
Poverty income ratio			0.853			0.003
0–1.29	2.18 (1.24, 3.83)	0.007		1.68 (1.02, 2.76)	0.042	
1.30–3.49	2.51 (1.57, 4.02)	<0.001		4.83 (3.13, 7.46)	<0.001	
≥3.50	2.8 (1.41, 5.55)	0.003		4.86 (2.64, 8.97)	<0.001	

Abbreviations: BMI, body mass index; HR, hazard ratio; SII, systemic immune‐inflammation index; SIRI, systemic inflammation response index.

However, we found that the risk of mortality was not consistently associated with increased SII or SIRI levels in some subgroups. For all‐cause mortality, the associations of the SII were nonsignificant in the age group of 20–59 years and uninsured individuals (*p* = .074 and *p* = .727, respectively). The association between SIRI and all‐cause mortality was also nonsignificant among uninsured patients (*p* = .396). Similarly, for cardiovascular mortality, nonsignificant relationships were observed for the SII in the less than 9th‐grade education subgroup and the SIRI in both the less than 9th‐grade education and uninsured subgroups (all *p* > .05).

Tests for interaction showed no significant subgroup effects in most analyses (P‐interaction >0.05). However, the P‐interaction was <0.05 for health insurance and PIR categories in all‐cause mortality analysis (P‐interaction = 0.004 and P‐interaction=0.011, respectively) and for PIR in cardiovascular mortality, indicating possible interaction effects (P‐interaction=0.003).

### 3.5 Sensitivity analyses

Recently, lower cutoff points for SUA levels (>5.6 mg/dL for men and >5.1 mg/dL for women) have been proposed to better identify hyperuricemia, as these thresholds are more closely related to cardiovascular disease outcomes.^19^ We performed a sensitivity analysis by redefining hyperuricemia based on these novel cutoff points, which resulted in 16,899 individuals being identified as having hyperuricemia. The weighted multivariate logistic analysis confirmed that SII and SIRI are predictive of both all‐cause and cardiovascular mortality in these patients (Table S1).

## 4 DISCUSSION

In this large prospective cohort study of US adults with hyperuricemia, we found that elevated levels of SII and SIRI were significantly associated with increased risks of all‐cause and cardiovascular mortality. The associations persisted after adjusting for demographic, socioeconomic, and clinical factors. RCS analyses revealed U‐shaped relationships between the SII and mortality outcomes and J‐shaped associations of the SIRI with mortality. Subgroup analyses did not identify significant effect modification. These findings provide novel evidence supporting the utility of the SII and SIRI as prognostic biomarkers among hyperuricemia patients who are at high risk for mortality.

In the evolving landscape of biomedical research, inflammation‐based prognostic scores such as the SII and SIRI have emerged as crucial tools. These markers, grounded on measurements of peripheral blood cell counts, provide an effective representation of the equilibrium between host inflammatory and immune status.^11^ A recent study found that the SII was positively associated with the SUA level in multivariate regression analysis among NHANES 2009–2018.^20^ Each 1‐SD increase in ln‐transformed SII was associated with a 38% higher risk of hyperuricemia. The associations were linear based on smooth curve fitting and stronger in adolescents ≥17 years.^20^ Earlier studies in a Chinese population indicated that SIRI was an independent predictor of hyperuricemia, with a 1‐SD increase in SIRI associated with 21.4% and 37.0% higher risk among men and women, respectively.^21^ These results provide evidence that the SII/SIRI may capture important inflammatory pathways linking elevated uric acid to adverse health outcomes.

Accumulating studies have evaluated the prognostic utility of the SII and SIRI in patients with diverse inflammatory conditions. Elevated SII and SIRI were independent predictors of worse outcomes in diseases such as sepsis, cancers, arteriosclerotic cardiovascular disease, COVID‐19, and even general populations.^13^, ^22^, ^23^, ^24^, ^25^, ^26^, ^27^ The consistent relationships with poor prognosis across various inflammatory diseases indicate that the SII and SIRI can serve as useful objective biomarkers reflecting systemic inflammation severity and relating to mortality risk.

However, to the best of our knowledge, no studies have examined the prognostic value of the SII and SIRI specifically in relation to hyperuricemia, despite experimental evidence suggesting that inflammation may be an important intermediary linking elevated uric acid to adverse health outcomes.^5^ Our study addressed this knowledge gap by investigating the SII and SIRI in a nationally representative cohort of US adults with hyperuricemia. The results align with prior research in general populations that found positive associations of the SII and SIRI with all‐cause and cardiovascular mortality.^13^ The higher HRs observed in our hyperuricemia cohort compared to previous studies in general populations further highlight the clinical relevance of incorporating the SII and SIRI for risk prediction in this high‐risk group.

Notably, we identified U‐shaped associations between the SII and mortality outcomes and J‐shaped relationships between the SIRI and mortality. The lower risks observed at moderate SII/SIRI levels may reflect normal baseline inflammatory status, while very high SII/SIRI indicates aggravated inflammatory burden with adverse prognosis. The nonlinear associations suggest caution in interpreting single cutoff‐based categories and support using the SII/SIRI as continuous variables in prediction models.

The strengths of this study include the population‐based cohort design, use of direct assay measurements for uric acid and leukocyte counts, long follow‐up duration, and robustness of results as demonstrated through sensitivity and subgroup analyses. An inherent limitation of observational studies is the potential for confounding factors to influence the observed associations. In our study, sex and age are significant confounders that must be carefully considered when interpreting the relationships between SII/SIRI and mortality risk among hyperuricemia patients. The incidence of hyperuricemia exhibits pronounced sex differences, with postmenopausal women having markedly increased prevalence and uric acid levels, while premenopausal women rarely develop this condition.^28^ Moreover, aging itself is closely associated with chronic, low‐grade systemic inflammation, known as “inflammaging,” which is a significant risk factor for various age‐related diseases, including cardiovascular diseases, diabetes, and neurodegenerative disorders.^29^ Although we adjusted for a series of covariates, including age and sex, in our regression models and conducted stratified subgroup analyses to mitigate potential confounding effects, we cannot definitively exclude residual confounding influences of these factors on the observed associations.

In conclusion, elevated SII and SIRI served as independent risk markers for all‐cause and cardiovascular mortality among US adults with hyperuricemia. These findings support incorporating the SII and SIRI in prognostic models to improve risk stratification in this high‐risk population. Future studies should validate these results and evaluate the utility of the SII/SIRI in guiding preventive interventions for hyperuricemia patients.

## AUTHOR CONTRIBUTIONS

**Tian Ren**: Data curation; Formal analysis. **Jian Wu**: Funding acquisition; Investigation. **Chao Wang**: Methodology; Software. **Yufeng Yin**: Conceptualization; Writing—original draft; Writing—review and editing.

## CONFLICT OF INTEREST STATEMENT

The authors declare that the research was conducted in the absence of any commercial or financial relationships that could be construed as a potential conflict of interest.

## Supporting information

Supporting information.

## Data Availability

All data generated and analyzed during this study are included in this published article.
