# Supplementary material for: Systemic inflammation markers independently associated with increased mortality in individuals with hyperuricemia: Results from the NHANES prospective cohort study
Source: Immun Inflamm Dis. 2024 Oct 1;12(10):e70032. doi: 10.1002/iid3.70032 (PMC11443515; doi:10.1002/iid3.70032)
Supplement: Supplementary file 1 — Supporting information. [file IID3-12-e70032-s001.docx]

**Supplementary materials**


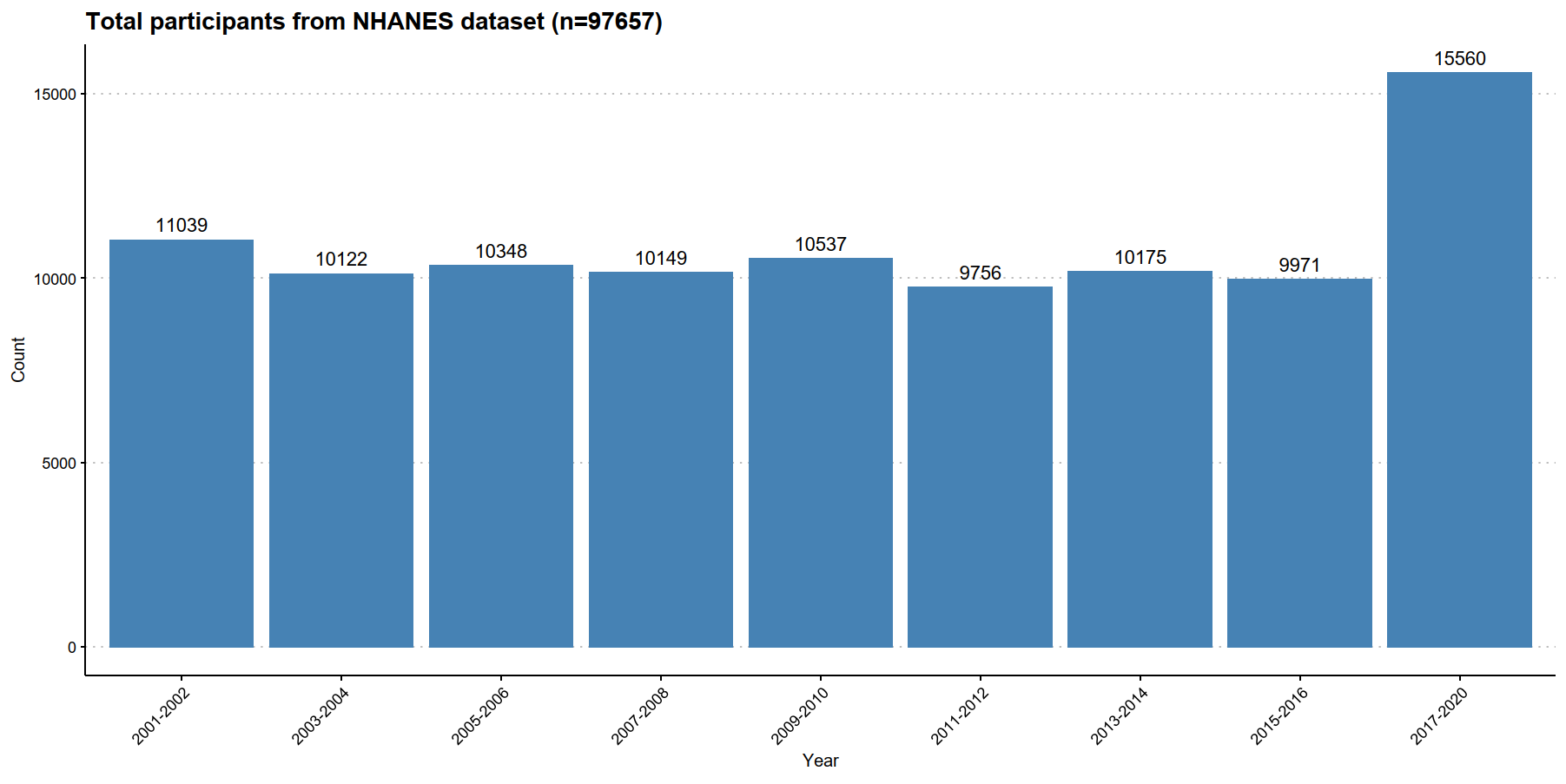


## Figure S1. Distribution of participants across different NHANES cycles.


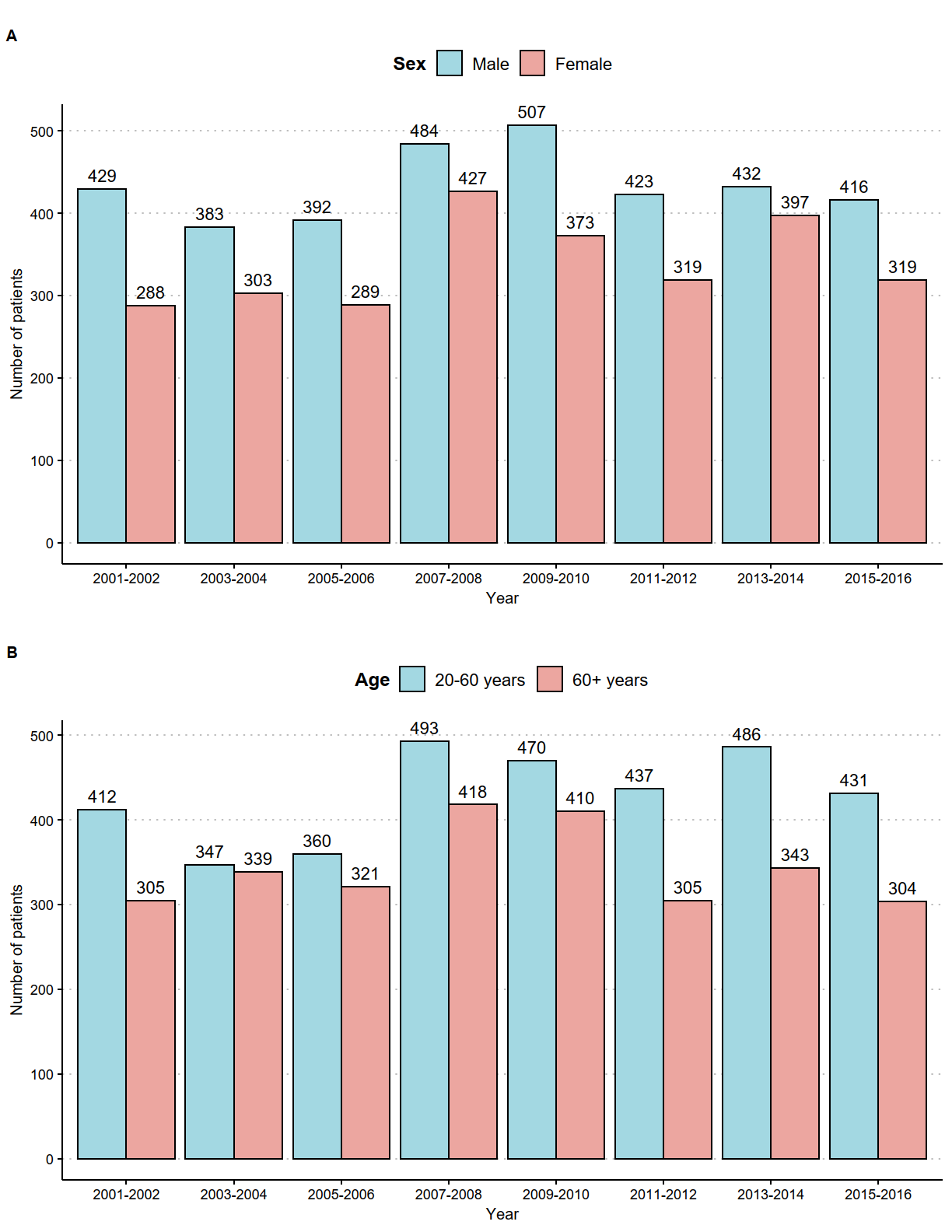

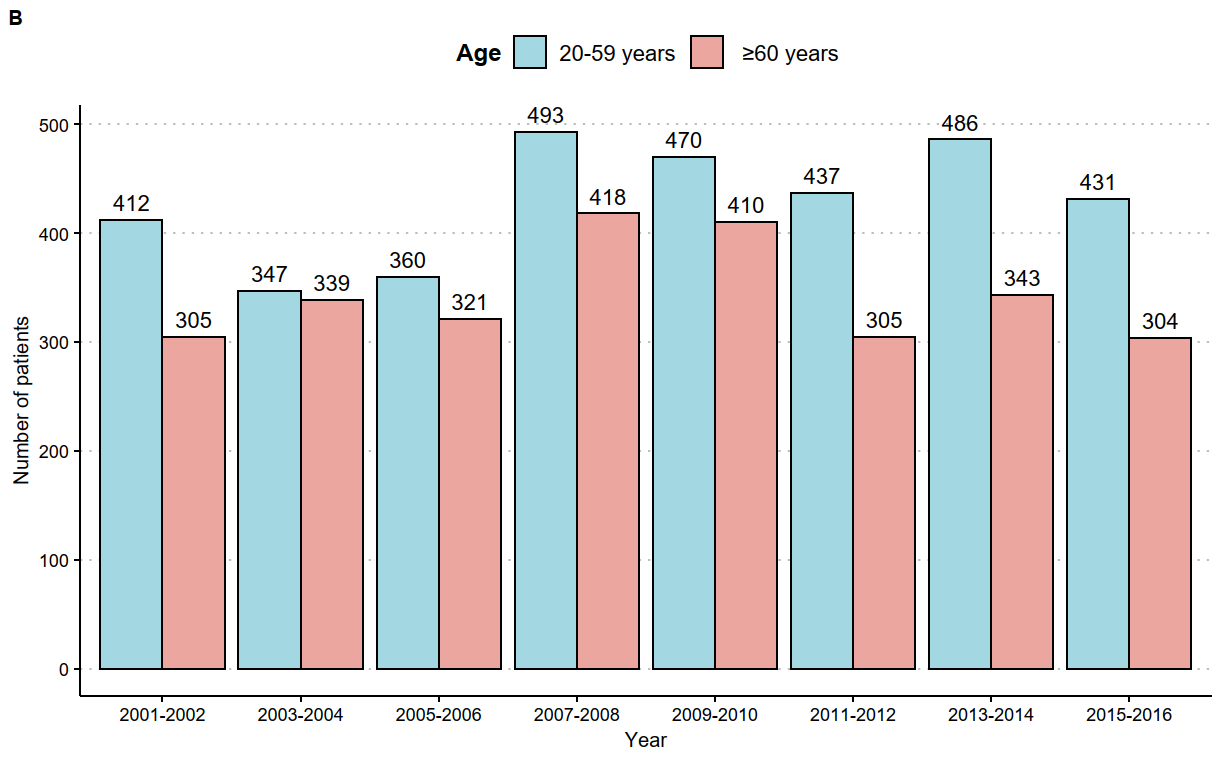


## Figure S2. Distribution of hyperuricemia patients (n=6181) across different NHANES cycles based on sex (A) and age (B) groups. Panel (A) shows the number of male and female patients, while panel (B) displays the number of patients aged 20-59 years and those aged ≥60 years in each cycle.

## Table S1. The predictive values of SII/SIRI for mortality in patients with hyperuricemia defined by lower SUA levels.

| **Characteristic** | **HR** | **95% CI** | **P value** |
| --- | --- | --- | --- |
| All-cause mortality |  |  |  |
| SII | 1.80 | 1.58, 2.05 | <0.001 |
| SIRI | 1.90 | 1.69, 2.14 | <0.001 |
| Cardiovascular mortality |  |  |  |
| SII | 2.05 | 1.45, 2.89 | <0.001 |
| SIRI | 2.01 | 1.57, 2.57 | <0.001 |

Note: The lower cut-off points of SUA for definition of hyperuricemia are 5.6 mg/dL for men and 5.1 mg/dL for women based on URRAH study. This reclassification resulted in 16,899 individuals being identified as having hyperuricemia. Weighted multivariate logistic analyses were performed based on Model 3, which was adjusted for age, sex, race/ethnicity, educational attainment, smoking status, BMI, health insurance status, and the poverty income ratio.

Abbreviations: SUA, serum uric acid; HR, hazard ratio; SII, systemic immune-inflammation index; SIRI, systemic inflammation response index; URRAH, Uric Acid Right for Heart Health.
